# A milestone in single-atom catalysis for direct formic acid fuel cell

**DOI:** 10.1093/nsr/nwaa228

**Published:** 2020-09-09

**Authors:** Jun Li

**Affiliations:** Department of Chemistry, Tsinghua University, China

Single-atom catalysts (SACs) have opened up an era of great opportunities for catalytic science and industrial technology, and have fueled the rapid progress of catalytic research [1–3]. SACs not only represent the ultimate atomic utilization efficiency of metal materials, but have also driven the quantitative-to-qualitative evolution of catalytic properties of materials brought about by size reduction. Thus, single-atom catalysis is a breakthrough in catalysis science towards atomically precise catalysis.

Among the applications of catalytic science, the fuel-cell car is one of the main competitors to the electric vehicle as an alternative to conventional petrol vehicles. For this revolutionary new technology, formic acid is a candidate as liquid fuel for direct formic acid fuel cells (DFAFC) because of its facile and safe transportation and storage. However, wide commercial application has been hindered by the lack of efficient and robust catalytic materials, particularly vis-à-vis the anodic formic acid oxidation reaction (FAOR). A breakthrough was made recently by Yadong Li and colleagues. They discovered a new class of catalysts (Rh_1_/CN and Ir_1_/CN) for formic acid oxidation [4,5], which show not only high mass activity but also remarkable stability. The Rh_1_/CN and Ir_1_/CN catalysts were discovered from a pool of SACs synthesized by a general `host-guest' strategy using ZIF-8 (zeolitic imidazolate framework) as a host to trap the metal precursors *in situ*. The obtained Rh_1_/CN and Ir_1_/CN materials had atomically dispersed metal sites and retained the porous structure and high specific surface area of the ZIF-8.

In contrast to Ir or Rh nanoparticles, which show negligible mass activity towards formic acid oxidation, Rh_1_/CN and Ir_1_/CN catalysts exhibit ultra-high mass activity, which is an order of magnitude higher than the state-of-the-art Pd/C catalyst. Moreover, these SACs also feature high resistance to CO poisoning as a result of huge endothermicity in CO absorption on single-atom Ir or Rh sites. Another exciting prospect of this work is that the mass power density of the integral fuel cell incorporated with Rh_1_/CN is higher than that of the Pd/C one, suggesting that these novel SACs have a bright future in practical fuel cell application.

From the theoretical viewpoint of breaking and forming chemical bonds, reduction of the metal size from nanoscale to single-atom scale is a process of transition from metal–metal bonding in bulk metals to metal–ligand covalent bonding in SACs. Analogous to the ligands of mononuclear metal homogeneous catalysts, the support of heterogeneous SACs containing abundant ligating atoms could be regarded as bulky heterogeneous ligands, which dictate the ligand field of the coordination environment and quantum states of the metal active center, and thus the catalytic properties [6–8]. There is room for further innovations in this affluent research field through joint experimental and theoretical efforts in rational design of heterogeneous ligands with unique coordination environments, general SACs fabrication strategies, and computational modeling and simulations.

***Conflict of interest statement.*** None declared.
